# Testing biasedness of self-reported microbusiness innovation in the annual business survey

**DOI:** 10.1371/journal.pone.0296667

**Published:** 2024-01-12

**Authors:** Luyi Han, Zheng Tian, Timothy R. Wojan, Stephan J. Goetz

**Affiliations:** 1 Northeast Regional Center for Rural Development, Penn State University, State College, Pennsylvania, United States of America; 2 Oak Ridge Institute for Science and Education Research Ambassadors Program, National Center for Science and Engineering Statistics, National Science Foundation, Oak Ridge, Tennessee, United States of America; 3 Northeast Regional Center for Rural Development, Department of Agricultural Economics, Sociology, and Education, Penn State University, State College, Pennsylvania, United States of America; UCL: University College London, UNITED KINGDOM

## Abstract

This study tests for potential bias in self-reported innovation due to the inclusion of a research and development (R&D) module that only microbusinesses (less than 10 employees) receive in the Annual Business Survey (ABS). Previous research found that respondents to combined innovation/R&D surveys reported innovation at lower rates than respondents to innovation-only surveys. A regression discontinuity design is used to test whether microbusinesses, which constitute a significant portion of U.S. firms with employees, are less likely to report innovation compared to other small businesses. In the vicinity of the 10-employee threshold, the study does not detect statistically significant biases for new-to-market and new-to-business product innovation. Statistical power analysis confirms the nonexistence of biases with a high power. Comparing the survey design of ABS to earlier combined innovation/R&D surveys provides valuable insights for the proposed integration of multiple Federal surveys into a single enterprise platform survey. The findings also have important implications for the accuracy and reliability of innovation data used as an input to policymaking and business development strategies in the United States.

## Introduction

Surveys of business innovation started in earnest in the U.S. in 2008 with the redesign of the Survey of Industrial Research and Development (SIRD), which became the Business R&D and Innovation Survey (BRDIS). Unlike the European Union’s Community Innovation Survey administered since 1992, BRDIS combined detailed questions on the R&D activities of firms with self-reported innovation questions regarding the introduction of new or significantly improved products and processes. A significantly lower incidence of innovation in the U.S. relative to most EU countries raised questions as to whether the difference may be due to a downward bias in innovation self-reports, consistent with U.S. respondents being more predisposed to viewing “new or significantly improved” as being only science- and engineering-based innovation. The issue became largely moot with the introduction of the Annual Business Survey (ABS) in 2018, in which firms with ten or more employees are only asked innovation questions, while R&D questions are asked separately in the Business Enterprise Research and Development Survey. However, microbusinesses (i.e., fewer than ten employees) in the ABS still receive both the R&D and innovation modules. Combined R&D and innovation survey, targeted to microbusinesses may lead to substantial underreporting of innovation activities if these businesses are less likely to pursue science and engineering based innovation. Indeed, microbusinesses report the lowest incidence of innovation in the ABS, raising the question of whether this is merely a function of firm size or a disadvantage attributable to survey design. We consider two types of product innovation reported in the ABS innovation module as dependent variables to test the potential bias. New-to-market product innovations represent new or significantly improved goods or services into the market before any competitors in the last three years, while new-to-business innovations represent new or significantly improved goods or services only new to firms but pre-existing in the market.

The administration of the inaugural ABS provides the information necessary to test for the bias attributable to this combined R&D and innovation survey design. A sharp threshold of ten employees was used by ABS administrators to partition the drawn sample into two groups–microbusiness and all other businesses, thereby determining which survey module(s) each group would receive. In other words, the ten-employee threshold was determined exogenously with respect to survey respondents, and all businesses should be accurately categorized into the two groups, considering microbusinesses as the treated and all other businesses as the controlled. These two conditions allow the use of a sharp regression discontinuity design to test the null hypothesis that there is no underreporting of innovation activities for firms on either side of the threshold. If the null hypothesis is not rejected with a statistically powerful test, our study will provide support for using the ABS data in microbusiness innovation studies. If the null is rejected, our study will caution researchers who want to use these data.

Previous literature discusses potential survey design biases that may impact self-reported innovation rates. Studies have found lower innovation rates when R&D questions are combined with innovation questions in the same survey compared to innovation-only surveys. Exact causes are unclear, but factors may include survey length, framing innovation as related to R&D, and title cues about survey topics. Other biases arise from survey mode and question formats [1, 2]. We review the empirical and cognitive testing literature addressing both the possible R&D bias in innovation surveys and the lower incidence of innovation in U.S. surveys relative to the EU Community Innovation Survey. A comparison of the BRDIS and ABS microbusiness surveys shows how the implicit conversation about innovation might be affected differently by how the R&D topic is introduced and organized. Details on the data are provided before specifying the regression discontinuity test. The findings are then discussed with respect to small business innovation research and implications are drawn for the proposed single enterprise platform in terms of the format or sequencing of unique modules to different types of firms.

The findings from this analysis have implications not only for designing innovation surveys but also for general survey planning by emphasizing the psychology of survey responses [3]. The vision for re-engineering Federal business surveys is to integrate multiple surveys into a single survey that businesses would be required to complete, reducing respondents’ burden with the objective of raising response rates [4]. For that purpose, the ABS “replaces the five-year Survey of Business Owners (SBO) for employer businesses, the Annual Survey of Entrepreneurs (ASE), the Business R&D and Innovation for Microbusinesses survey (BRDI-M), and the innovation section of the Business R&D and Innovation Survey (BRDI-S).” It was conceived as an interim step between multiple, independent surveys and the future single platform for businesses reporting administrative records and responding business surveys. Evidence that responses in one part of the ABS are influenced by other modules would raise serious questions regarding data quality in integrated surveys. Alternatively, evidence that the R&D module in the ABS does not bias innovation results for those respondents who receive it may provide valuable information for the modular design of integrated surveys. Does the inclusion of an R&D module for microbusiness with 10 or fewer employees cause potential bias in self-reported innovation in the ABS survey? We use a regression discontinuity design (RDD) to assess the bias.

## Literature review

The study most resembling the objectives of this article was conducted by Statistics Norway. Previously, the U.S. and Norway both had self-reported innovation rates considerably below those of their EU peers. Innovation surveys in both countries were mandatory, and both combined R&D and innovation activities. In contrast, most of the innovation surveys from peer EU countries are voluntary and contain few questions pertaining to science and engineering-based innovation and R&D. The seemingly poor innovation performance—using self-reported innovation questions as suggested by the Oslo Manual [1]—of two of the most economically advanced countries in the world provided the motivation for examining potential bias in combined R&D/innovation surveys rigorously. Norway modified the 2011–2012 administration of their national Innovation in Norwegian Business survey by designating two special subsets of firms that were sent innovation-only surveys similar to the EU’s Community Innovation Survey [2]. One set was required to complete the survey while completion was voluntary for the other set. Product or process innovation rates were highest in the voluntary, innovation-only survey (41%); lower in the mandatory, innovation-only survey (30%); and lowest in the standard mandatory, R&D/innovation survey (19%). To the extent that all three samples were representative of Norwegian businesses, the exercise confirmed the tendency for R&D/innovation surveys to find lower rates of self-reported innovation relative to innovation only surveys. However, because the respondents in each sample were different it was not possible to identify respondents’ characteristics or response behaviors that may explain the greater reluctance of R&D/innovation survey respondents to self-identify as innovators.

Tian, Wojan, and Goetz [5] attempted to compare responses from the same firm to an innovation-only and combination innovation-R&D survey by linking the 2014 BRDIS to the 2014 Annual Survey of Entrepreneurs (ASE)—the precursor to the ABS that also included an innovation module but no R&D module. Despite seemingly promising sample sizes of approximately 290,000 for ASE and 44,000 for BRDIS, the resulting number of matches was limited and could not support a formal analysis of survey response variation. In addition to the small sample size, additional error would be introduced by the possibility that surveys were completed by different people or different business units within the organization, further reducing the ability to discern meaningful differences from the same firm responding to different surveys. Square table analysis used to assess the strength of agreement of responses by matched firm responses was largely uninformative. However, logistic regression of the pooled BRDIS-ASE data did indicate that BRDIS respondents were more than twice as likely to report no product innovation as ASE respondents after controlling for industry and firm size, reinforcing the findings from Wilhelmsen [2].

A conference paper by Hoskens et al. [6], presented at the OECD Blue Sky Forum on Science and Innovation Indicators, discussed various method effects that have been associated with differences in the incidence of innovation for surveys conducted in the same country. In addition to the effect of R&D modules lowering innovation rates identified by Wilhelmsen [2], other method-related effects result from the use of long vs. short innovation surveys [6, 7] and differences in the mode of data collection [8]. Shifting from a paper survey to a web survey containing one question per screen seemingly lessened the tendency to report no innovation as a means of avoiding follow-up questions [8].

*The Psychology of Survey Response* [3] provides a rich framework for assessing how survey design decisions might introduce sources of potential bias. The main insight from this approach is that the intended purpose of surveys, i.e., to collect objective information, is not always the criterion that respondents use in answering questions. A survey also represents a conversation between respondents and enumerators (or survey instruments), so differences in response may be due to survey mode, short or long version of completion, and survey titles or headings that may influence various aspects of the implicit conversation. In the context of innovation surveys, this may be manifest in the decision to self-report “new or significantly improved” products or processes.

Analyzing the possible impact of method or survey design choice on reported innovation rates within a regression discontinuity framework highlights the most critical aspects of a treatment: its dosage. Within ABS, the inclusion or omission of the R&D module is binary depending on whether the firm employed fewer than ten employees. However, the effect of the R&D module in ABS may be quite different from the effect of the R&D module on innovation questions in BRDIS vis-à-vis the innovation-only questions in ASE. A closer look at how the R&D topic is introduced and covered in ABS and BRDIS is necessary to inform our priors on the size of the effect we expect to detect.

The most obvious difference is the title of the surveys. Respondents to the Business R&D and Innovation Survey know what “the conversation” is likely to be about. Respondents to the Annual Business Survey are given no such clues and thus are less likely from the outset to conclude that the survey is or is not relevant to their firms. Firms engaged in grassroots innovation may choose to opt out of full participation in BRDIS, and thus may fail to appreciate that the innovation questions are independent of R&D activity. The dosage of the R&D module treatment in BRDIS can be thought of as much larger and more front-loaded than the dosage of the treatment in ABS at the outset.

**Fig 1:**
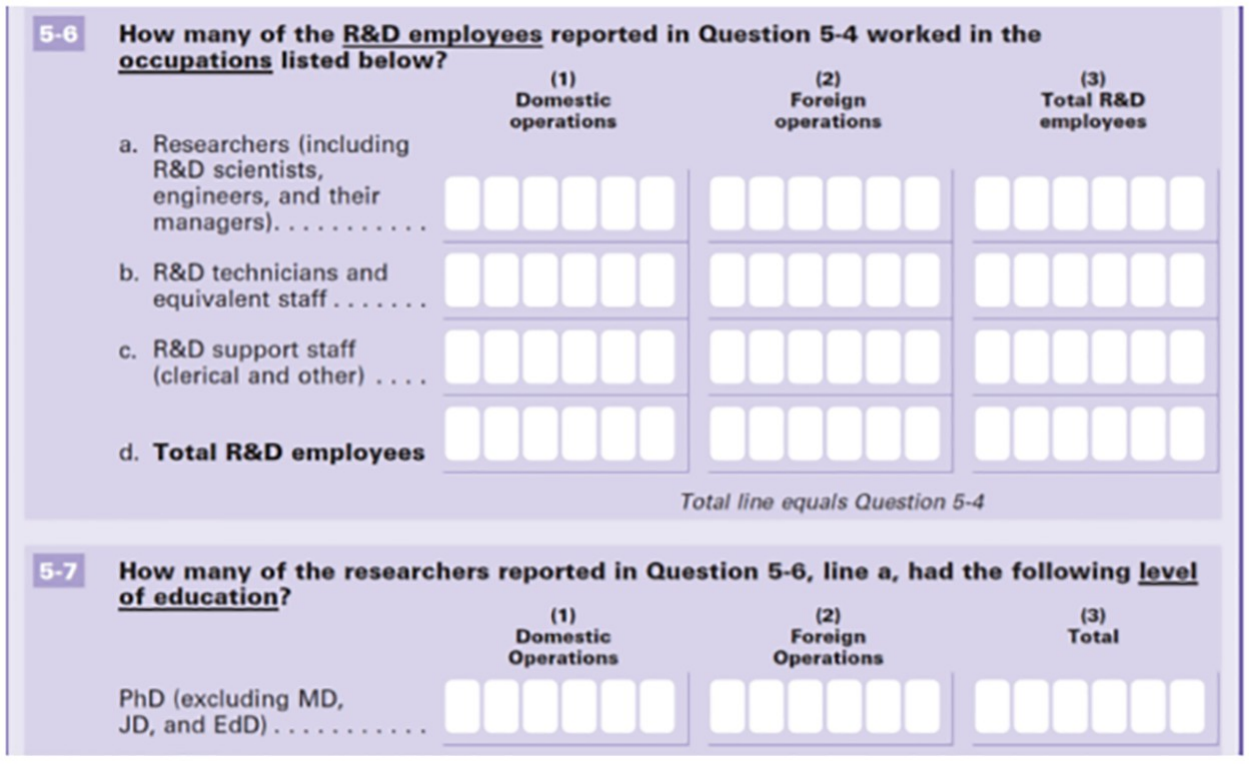
Screenshot of human resource questions from 2016 Business R&D and innovation survey. Notes: 2016 BRDIS [survey instrument] available at https://www.nsf.gov/statistics/srvyindustry/about/brdis/surveys/srvybrdis-2016-BRDI-1.pdf.

The innovation questions precede the R&D questions in both surveys to help minimize the bias caused by framing innovation as that directly related to science and engineering. For respondents who only interact with the online survey, their initial response to the innovation questions may be little influenced by the R&D questions. The big difference between BRDIS and ABS is the number of R&D questions received by R&D performers and nonperformers. In BRDIS, firms that indicate no formal R&D budget skip the financial questions but are still asked questions about R&D human resources, e.g., how many scientists and engineers are employed by the company, and how many have PhDs (Fig 1). In contrast, R&D nonperformers in ABS skip all questions related to R&D finances and R&D human resources. BRDIS respondents who are not R&D performers may be inclined to revisit the innovation questions after considering the R&D human resource questions. Of course, the inclusion of R&D questions may influence all respondents who view the entire “Information Copy” of the surveys before completing the survey online. But only BRDIS respondents are required to read and answer questions on R&D human resources at their firms through the online survey instrument. This again would suggest that the dosage of R&D questions in BRDIS may bias responses relative ABS.

A final consideration of the treatment dosage that may affect a firm’s revelation about its innovation activities is the relative centrality of the innovation and R&D topics to the conversation. Because ABS combines the former Survey of Business Owners (SBO) with the innovation questions from BRDIS and microbusiness R&D questions from the former Business R&D and Innovation Microbusiness survey (BRDI-M), the conversation starts much differently than the conversation in BRDIS. After the standard questions confirming the continuity and ownership structure of the business, ABS elicits information on the business owners: their age, prior business ownership experience, educational background, and reasons for starting or acquiring the business. In this regard, ABS is much better at “getting to know the respondent,” particularly for microbusinesses where an owner is likely to be the person completing the innovation and R&D sections that follow. This may produce a higher comfort level than in BRDIS, where the innovation questions are the first substantive questions received. Firms not engaged in R&D may be particularly reticent to respond affirmatively to these initial innovation questions given the cue from the survey title that R&D questions will follow.

The comparison of responses to innovation questions in BRDIS and ASE by Tian, Wojan, and Goetz [5] provides an upper bound for the effect size of the downward bias that might be attributable to the inclusion of an R&D module in an innovation survey. The anticipated effect size for ABS respondents on either side of the 10-employee threshold should be significantly lower than that found in the BRDIS-ASE study given that R&D nonperformers have minimal exposure to the R&D questions and the survey title does not cue up R&D as a questionnaire topic. It is difficult to assess the extent to which the differences between the BRDIS and ABS survey instruments affect the psychology of responding to self-reported innovation question. However, a lower bound of less than one-quarter the ostensible BRDIS effect would explain the observed difference in innovation rates between microbusinesses (< 10 employees) and the smallest business size class over the threshold (10–19 employees) which is 2.3% [9]. The power of the regression discontinuity test for detecting small effects will thus be essential for assessing the robustness of findings and the extent to which the results may be due to measurement error.

In sum, this comprehensive examination of innovation surveys, particularly in the context of R&D module inclusion, underscores the complexity of measuring self-reported innovation rates accurately. The research conducted by Statistics Norway, as well as other relevant studies, highlights the potential for survey design choices and methodological differences to impact the reported incidence of innovation within a given country. From the mandatory vs. voluntary survey participation to the framing of questions and the dosage of the R&D module, each element may contribute to nuances in survey response. Moreover, the inherent conversational nature of surveys, influenced by survey titles and respondent characteristics, further complicates the interpretation of self-reported innovation data. In our study, we use the ABS data to test the null hypothesis that there is no underreporting of innovation activities due to the survey design in which microbusinesses receive both R&D and innovation modules, while small businesses (10 or more employees) receive only an innovation module. Additionally, our study primarily concentrates on businesses with approximately 10 employees, thereby contributing to the literature on small business innovation and entrepreneurship [10–13].

### Data

We use two confidential Census datasets, accessed through an approved research project at a Federal Statistical Research Data Center (FSRDC). The 2018 Annual Business Survey, for which 2017 is the reference year, contains self-reported innovation, industry (defined by the North American Industry Classification System or NAICS), and firm size. We also use the 2016 Longitudinal Business Database (LBD), of which firm records came from the 2016 Business Register (BR) that were used to draw the 2018 ABS sample. According to US Census Bureau, “Annual Business Survey data are sourced from a combination of responses to the survey, data from the economic census, and administrative records data. The ABS is primarily collected using an electronic instrument. The survey was mailed to approximately 850,000 employer businesses. Businesses selected to report were sent a letter informing of their requirement to report… Approximately 67.8 percent of the sample responded to the survey.” Employment size in the BR determined which firms received the microbusiness (<10 employees) survey containing the innovation and R&D modules and which receiving the innovation-only survey. Although the 2018 ABS microdata also contains firms’ employment in 2017, we are concerned that these employment data may not equal the 2016 BR data as firms may have grown or shrunk during these two years. Therefore, we use the 2016 LBD employment data as the running variable in regression discontinuity design, while using the 2018 ABS data for all other firms’ and innovation related information.

Microbusinesses in the 2018 ABS received the ABS-1 survey instrument, while all other firms received the ABS-2 instrument. Table 1 shows the summary statistics for all firms in the two groups (Panel A) as well as firms around the 10-employee threshold with half, one, and two bandwidths (Panels B to D, respectively). The bandwidth selection is explained in the next section. Each panel shows summary statistics separately for firms below or above the 10-employee cutoff. We use the ABS sample weights to compute the summary statistics. We do not report the minimum or maximum values of variables following FSRDC disclosure avoidance rules. In the full sample reported in Panel A, the average employment size across all samples is about eight, the average employment size of microbusinesses is three below the cutoff, and 30 for all other firms above the cutoff.

**Table 1:** Summary statistics.

	Variable	N	Mean	Std. Err
**Panel A: Full Sample**				
firmsize_emp less than 10	firmsize_emp	334000	2.932	0.004176
	New-to-market	334000	0.08447	0.000481
	New-to-market fitted	334000	0.09304	8.18E-05
	New-to-business	334000	0.1624	0.000638
	New-to-business fitted	334000	0.1696	9.03E-05
firmsize_emp equal or larger than 10	firmsize_emp	130000	29.86	0.214
	New-to-market	130000	0.104	0.000845
	New-to-market fitted	130000	0.1012	0.000144
	New-to-business	130000	0.1789	0.001061
	New-to-business fitted	130000	0.1747	0.000149
Total	firmsize_emp	464000	8.317	0.05321
	New-to-market	464000	0.08838	0.000417
	New-to-market fitted	464000	0.09469	0.000071
	New-to-business	464000	0.1657	0.000546
	New-to-business fitted	464000	0.1707	7.71E-05
**Panel B: Half Bandwidth**				
firmsize_emp less than 10	firmsize_emp	11000	9	0
	New-to-market	11000	0.0962	0.002819
	New-to-market fitted	11000	0.09892	0.000473
	New-to-business	11000	0.1745	0.003628
	New-to-business fitted	11000	0.1745	0.000496
firmsize_emp equal or larger than 10	firmsize_emp	17500	10.45	0.003762
	New-to-market	17500	0.09951	0.002263
	New-to-market fitted	17500	0.09936	0.000374
	New-to-business	17500	0.179	0.002899
	New-to-business fitted	17500	0.1744	0.000389
Total	firmsize_emp	28500	9.881	0.00479
	New-to-market	28500	0.09821	0.001765
	New-to-market fitted	28500	0.09918	0.000293
	New-to-business	28500	0.1772	0.002264
	New-to-business fitted	28500	0.1744	0.000306
**Panel C: One Bandwidth**				
firmsize_emp less than 10	firmsize_emp	40000	7.858	0.00403
	New-to-market	40000	0.09427	0.001458
	New-to-market fitted	40000	0.09732	0.000241
	New-to-business	40000	0.1733	0.001888
	New-to-business fitted	40000	0.1732	0.000254
firmsize_emp equal or larger than 10	firmsize_emp	31000	11.3	0.006327
	New-to-market	31000	0.1019	0.001725
	New-to-market fitted	31000	0.09918	0.000283
	New-to-business	31000	0.1781	0.002182
	New-to-business fitted	31000	0.1739	0.000296
Total	firmsize_emp	71000	9.288	0.00729
	New-to-market	71000	0.09744	0.001113
	New-to-market fitted	71000	0.09809	0.000183
	New-to-business	71000	0.1753	0.001427
	New-to-business fitted	71000	0.1735	0.000193
**Panel D: Two Bandwidths**				
firmsize_emp less than 10	firmsize_emp	154000	5.117	0.004827
	New-to-market	154000	0.08985	0.000729
	New-to-market fitted	154000	0.09526	0.000121
	New-to-business	154000	0.168	0.000953
	New-to-business fitted	154000	0.1714	0.000129
firmsize_emp equal or larger than 10	firmsize_emp	49000	12.73	0.01001
	New-to-market	49000	0.1024	0.001366
	New-to-market fitted	49000	0.0994	0.000226
	New-to-business	49000	0.1803	0.001733
	New-to-business fitted	49000	0.1737	0.000237
Total	firmsize_emp	203000	6.774	0.008229
	New-to-market	203000	0.09258	0.000643
	New-to-market fitted	203000	0.09616	0.000107
	New-to-business	203000	0.1707	0.000835
	New-to-business fitted	203000	0.1719	0.000113

Notes: 2018 ABS and 2016 LBD.

We consider two types of product innovation reported in the ABS innovation module for both treatment and control groups. New-to-market product innovations represent new or significantly improved goods or services into the market before any competitors in the last three years, while new-to-business innovations represent new or significantly improved goods or services only new to firms but pre-existing in the market. As shown in Table 1, for the two innovation variables, new-to-market and new-to-business innovation dummies, we in general see a higher incidence of innovation above the cutoff. For example, the average new-to-market innovation rate is around 8 percent below the cutoff, and over 10 percent above the cutoff. In Panels B, C, and D, we report similar statistics using different RDD bandwidths.

## Models and methods

We use a sharp regression discontinuity design (RDD) to assess the effect of distributing different formats of the ABS survey instruments to firms with more or less than 10 employees on self-reporting new-to-market and new-to-business innovations. The running variable (Xi) in the sharp RDD is a firm’s employee size and the cut-off value (c) is 10. The outcome variable (Yi) is a binary innovation variable. A sharp RDD is appropriate because microbusinesses with fewer than 10 employees would mandatorily receive the ABS-1 survey instrument containing both the R&D and innovation modules, while firms with 10 or more employees would receive the ABS-2 instruments without the R&D module. Although it is natural to think of microbusinesses as the treatment group as they received “additional” survey questions, to conform with the RDD literature in which a treated unit is the one with the running variable above the cutoff, we consider microbusinesses as the control group and other firms as the treatment group. The assignment of the treatment occurs ex ante ensuring that it is independent of the outcome variable.

A typical sharp regression discontinuity design considers the expected difference in the potential outcome when the running variable takes a value immediately above or below the cut-off value. That is, the sharp RDD considers the local average treatment effect (LATE) at the cut-off value, which can be expressed as [14]

$$\tau_{SRD}=\underset{X_{i}↓c}{\text{lim}}E\left(Y_{i}|X_{i}=c\right)-\underset{X_{i}↑c}{\text{lim}}E\left(Y_{i}|X_{i}=c\right)$$ (1)

The sharp RDD can also be written simply as a linear regression model [15]

$$Y_{i}=\alpha +\tau D_{i}+\beta (X_{i}-c)+\epsilon_{i}$$ (2)

where *D*_*i*_ = 1 if *X*_*i*_ ≥ c or *D*_*i*_ = 0 if *X*_*i*_ < *c*. Eq (2) can be estimated with either global or local least squares estimation. The global estimation uses all samples, and the local estimation uses samples delineated by a bandwidth around the cut-off value. The bandwidth selection is based on the mean-squared-error (MSE) optimal bandwidth choice method as explained in Cattaneo and Titiunik [16].

Regression discontinuity designs are used for causal inference in many fields of economics. A comprehensive review of RDD can be found in papers of Cattaneo and Titiunik [16], Imbens and Lemieux [14], and Lee and Lemieux [17]. The applications cover such topics as education [18, 19], health [20], family [21], and relevant to our paper, firm innovation [22–24]. Bronzini and Piselli [23] examine the effect of Italian regional government subsidies on R&D output measured by the number and the probability of patent applications. In their sharp RDD strategy, the dependent variables are either discrete count data for the number of patent application or a binary data for whether to file patent applications. They subsequently use the Poisson model in the RDD for the count variable and the logit model for the binary variable and estimate the models with all samples or selected samples defined by some bandwidth. Their estimation strategy is similar to ours since we also use a binary dependent variable and estimate the logit model in the RDD with various sample ranges.

Based on the generic model in Eq (2), we estimate an expanded linear regression model indexed for firm *i*:

$$Innovation_{i}=\alpha +\tau D_{i}+\beta (Emp_{i}-10)+\gamma (Emp_{i}-10)\times D_{i}+\rho X_{i}+\;\epsilon_{i}$$ (3)

The dependent dummy variable *Innovation*_*i*_ captures new-to-market innovations and new-to-business innovations. The S1 Appendix shows how these two variables are defined. *D*_*i*_ is the treatment variable where *D*_*i*_ = 1 if firms have equal to or more than ten employees. (*Emp*_*i*_ − 10) is the normalized running variable for employee counts. We also include an interaction term of the treatment and the normalized running variables to allow the slopes to vary on both sides of the cutoff point. *X*_*i*_ embeds the firm level control variables, including the highest education of the firm owner (Jankowski, et al. forthcoming), and the NAICS 2-digit industry dummies.

The outcome variable in a sharp RDD is usually a continuous variable. However, our dependent variable is binary. So, we estimate the model in three ways. First, we use the binary dependent variable as it is and interpret the model as a linear probability model (LPM). Second, we estimate a logit model for Eq (3) with *Y*_*i*_ transformed with the log(*u*/(1-*u*)), where *u* = Pr(*Y*_*i*_ = 1). Third, we first estimate a probit model for the binary outcome variable on education and NAICS dummies and then use the fitted value of the probit model as the outcome variable in Eq (3). The fitted value regressions do not include education and NAICS dummies as they are used in the first stage probit model.

As noted above, the RDD estimates LATE around the cutoff point. That is, we test the null hypothesis, *H*_0_: τ = 0 and γ = 0, around the ten-employee cutoff in the proximity determined by the optimal bandwidth. For example, if the optimal bandwidth is four, we estimate the RDD regressions only based on firms from which their employment sizes are between 6 and 14. We use the mean-squared-error (MSE) optimal bandwidth choice method to determine the bandwidth [25]. We use Stata command “rdbwselect” with “mserd” option to choose the optimal bandwidth. We also report estimates using half bandwidth and two bandwidths as robustness checks.

An important assumption affecting RDD validity is the absence of manipulation around the RDD cutoff point. In this analysis, it means firms are not able to overreport their employees (≥ 10 employees) strategically in order to skip the R&D module. Such a time-saving strategy would be most valuable to R&D performing microbusinesses, firms that tend to have a very high incidence of innovation [26]. This strategic behavior would bias innovation estimates below the threshold downward and estimates above the threshold upward. However, this manipulation is impossible because assignment to the ABS-1 or ABS-2 survey is based on administrative data rather than self-reported employment.

To address statistical power concerns owing to reliance on a small subsample of the data in an attempt to detect a small effect, we also provide RDD power tests to evaluate both the robustness of our findings along with the validity of inferences from negative findings [27]. We use Stata command “rdpow” to conduct RDD power tests. The approach is based on the continuity and smoothness assumptions and typically uses local polynomial methods.

## Results

As the ABS and LBD data are confidential, only accessible inside an FSRDC, our results have undergone a disclosure review process, including rounding of coefficients, standard errors, and observations according to disclosure avoidance rules.

### RDD figures

**Fig 2:**
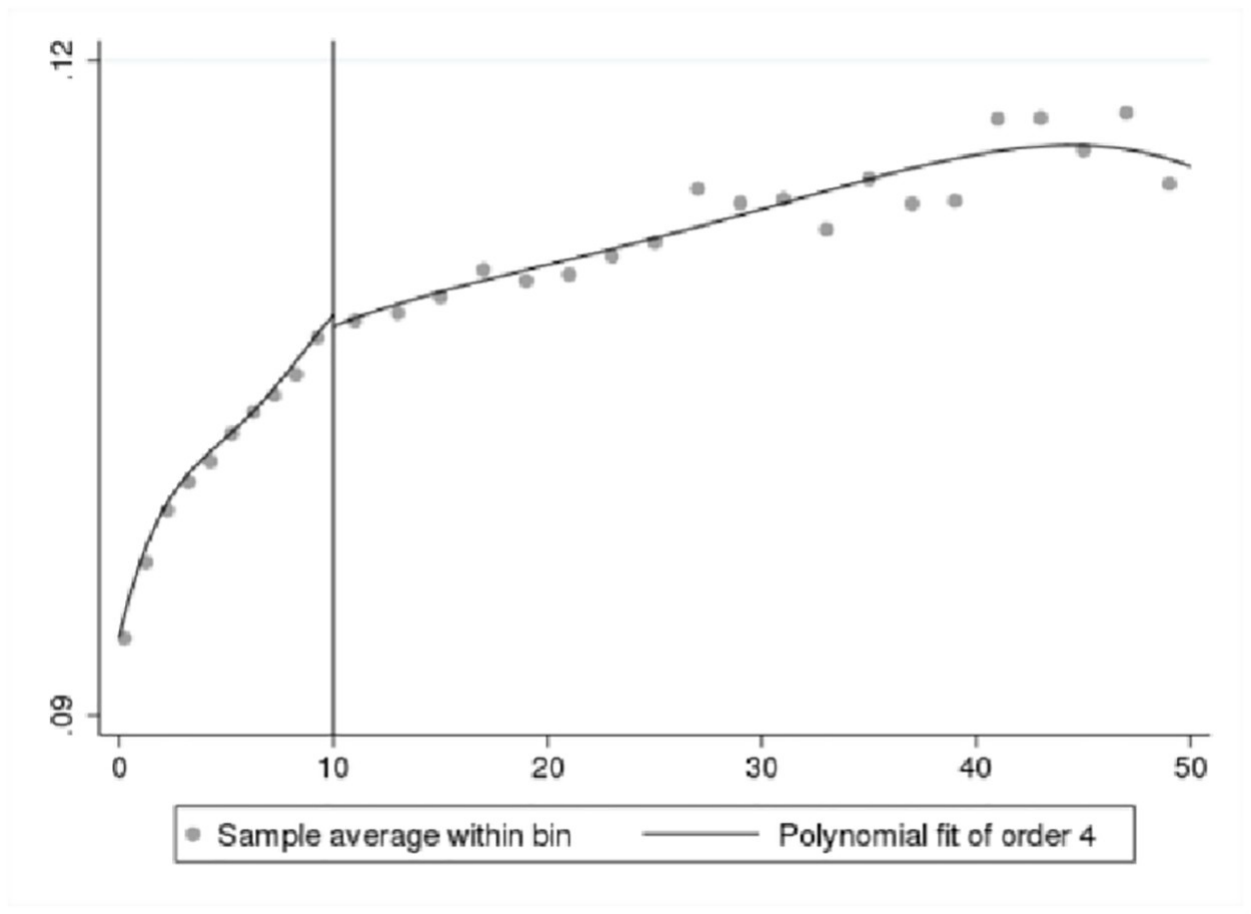
RDD plot for fitted new-to-market innovation. Notes: ABS 2018 and LBD 2016, authors’ calculations.

**Fig 3:**
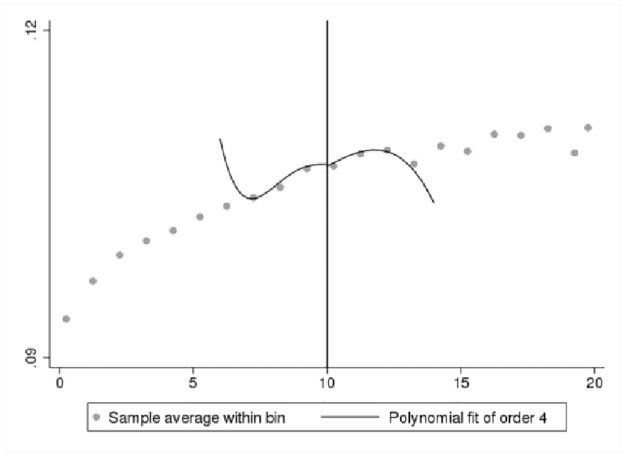
RDD plot for fitted new-to-market innovation restricted to bandwidth. Notes: ABS 2018 and LBD 2016, authors’ calculations.

**Fig 4:**
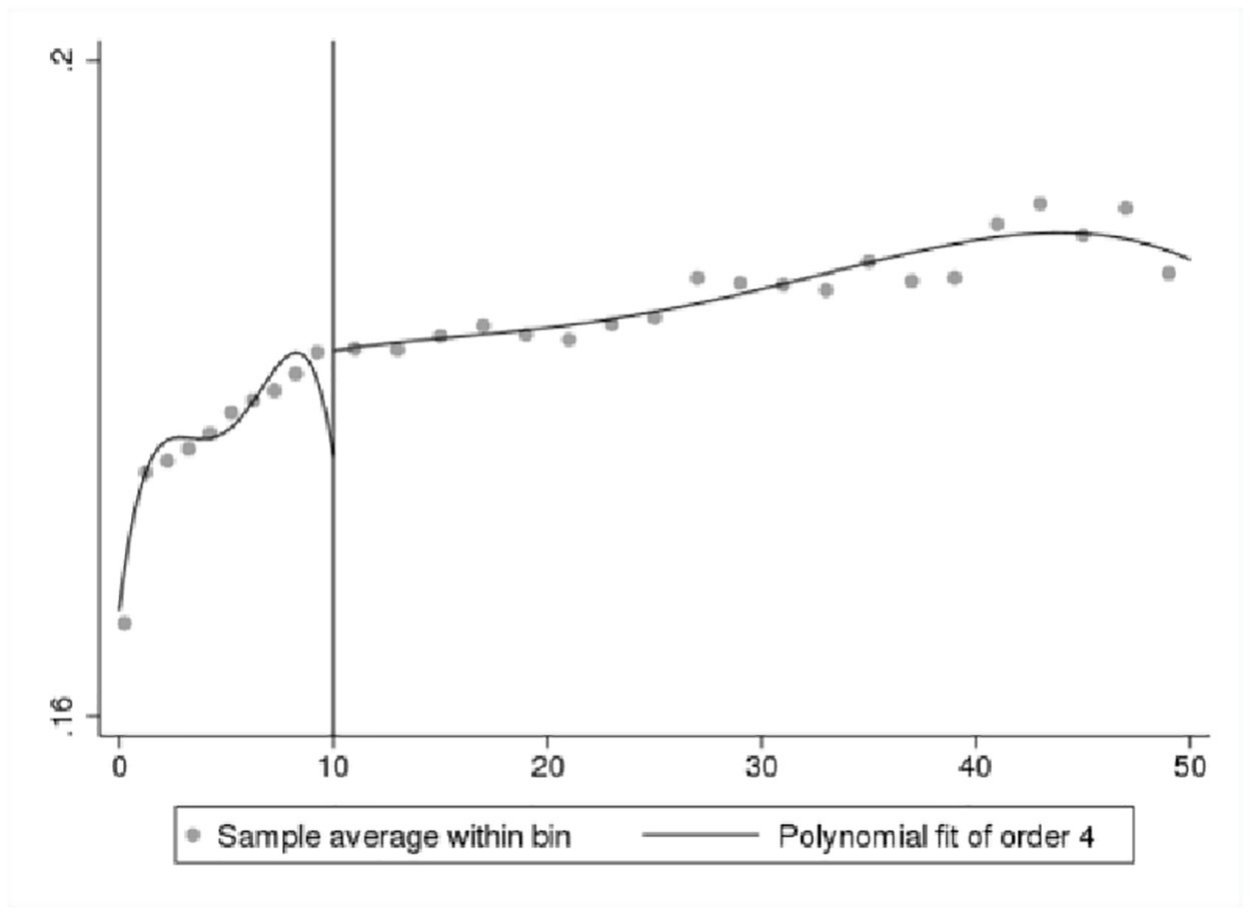
RDD plot for fitted new-to-business innovation. Notes: ABS 2018 and LBD 2016, authors’ calculations.

**Fig 5:**
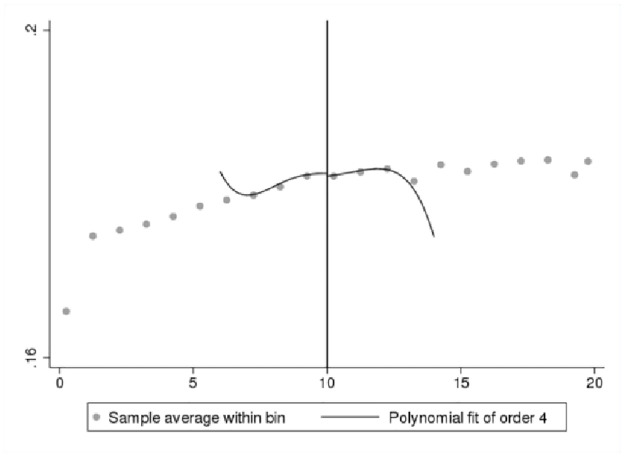
RDD plot for fitted new-to-business innovation restricted to bandwidth. Notes: ABS 2018 and LBD 2016, authors’ calculations.

We first present a set of data-driven RDD plots to provide preliminary visual evidence of the existence of a jump in innovation self-reports at the 10-employee cutoff. We use Stata command “rdplot” to generate RDD figures. We only plot the fitted values generated from a probit model for the binary outcome as discussed in Eq (3). In Figs 2–5, the X-axis shows bins by the number of employees, and the Y-axis shows the fitted values for the binary innovation outcome variables. We specify the fourth order of the polynomial fit to approximate the population conditional expectation functions for the control and treated units [28]. We show two types of figures for the fitted new-to-market and new-to-business innovation variables. One is a fitted line for the first 50 employment size bins, and another is restricted by the RDD bandwidth. Overall, Figs 2–5 demonstrate a smooth transition in innovation self-reports at the 10-employee cutoff.

### RDD regressions

Tables 2 and 3 show the main RDD regressions for new-to-market innovation and new-to-business innovation, respectively. We report three panels in each table, Panel A shows the LPM regression for the binary innovation variable, Panel B shows the logit regression, and Panel C shows the probit regression for fitted values. For each column in these two tables, column (1) reports the global regression using all firms, column (2) restricts the sample on the half bandwidth, column (3) uses the full bandwidth, and column (4) uses two bandwidths. In Table 2 Panel A, column (1) shows a 0.007 statistically significant point estimate on the RDD treatment variable, but coefficient estimates in the remaining columns (2), (3), and (4) are not statistically significant at the 10% level. Since the RDD method only looks at the causal effects around the cutoff (LATE), the results from the last three columns are most informative. They all suggest no statistical difference in self-reported innovation below or above the 10-employee cutoff, implying that we are unable to detect substantial survey bias among microbusinesses (fewer than 10 employees) answering R&D and innovation modules relative to all other businesses that did not receive an R&D module. A more definitive statement requires an estimate of the probability of detecting a substantial bias if true, which is provided in the section below. The positive and statistically significant effects in column (1) confirms the common finding that larger firms are more likely to report innovation across the complete sample. Results from using logit or probit fitted outcome variables in Panels B and C are consistent with our findings from Panel A. They all suggest no significant effects at the cutoff. Table 3 reports the new-to-business innovation variable, and we find similar results as Table 2. In Tables 2 and 3, the interaction term in column (2) gets dropped from the model automatically. It may be because the narrowest bandwidth effectively reduces the normalized running variable to a dummy. Then, the interaction term of the RDD treatment and the normalized running variable is also a dummy. For sensitivity analysis, the results remain robust when omitting the interaction terms.

**Table 2:** RDD for new-to-market innovation.

	(1)	(2)	(3)	(4)
**Panel A: LPM Model of Binary Y**				
treated10 = 1	0.0074	-0.0069	0.0012	0
	(p < 0.001)	(0.382)	(0.814)	(1.000)
firmsize_emp_rd10	0.0011	0.0043	0	0.0013
	(p < 0.001)	(0.360)	(1.000)	(0.001)
1.treated10#c.firmsize_emp_rd10	-0.0011		0.0026	-0.0004
	(p < 0.001)		(0.298)	(0.568)
Observations	464000	28500	71000	203000
Sample	all	0.5 bandwidth	bandwidth	2 bandwidth
**Panel B: Logit Model of Binary Y**				
treated10 = 1	0.0662	-0.0706	0.0129	-0.0019
	(p < 0.001)	(0.388)	(0.811)	(0.953)
firmsize_emp_rd10	0.014	0.0442	0	0.0147
	(p < 0.001)	(0.365)	(1.000)	(p < 0.001)
1.treated10#c.firmsize_emp_rd10	-0.0138		0.0266	-0.0058
	(p < 0.001)		(0.304)	(0.451)
Observations	464000	28500	71000	203000
Sample	all	0.5 bandwidth	bandwidth	2 bandwidth
**Panel C: Probit Fitted Y**				
treated10 = 1	0.0023	-0.0008	-0.0004	0
	(p < 0.001)	(0.568)	(0.657)	(1.000)
firmsize_emp_rd10	0.0014	0.0011	0.0013	0.001
	(p < 0.001)	(0.169)	(p < 0.001)	(p < 0.001)
1.treated10#c.firmsize_emp_rd10	-0.0014		-0.0011	-0.0009
	(p < 0.001)		(0.006)	(0.003)
Observations	464000	28500	71000	185000
Sample	all	0.5 bandwidth	bandwidth	2 bandwidth

Notes: 2018 ABS and 2016 LBD. Column (1) reports the global regression using all firms, column (2) restricts the sample on the half bandwidth, column (3) uses the full bandwidth, and column (4) uses two bandwidths. P-values are in parentheses.

**Table 3:** RDD for new-to-business innovation.

	(1)	(2)	(3)	(4)
**Panel A: LPM Model of Binary Y**				
treated10 = 1	0.0126	-0.0034	0.0057	-0.0018
	(p < 0.001)	(0.729)	(0.373)	(0.636)
firmsize_emp_rd10	0.0008	0.0062	-0.001	0.0016
	(0.008)	(0.293)	(0.677)	(0.001)
1.treated10#c.firmsize_emp_rd10	-0.0007		0.0019	0.0006
	(0.020)		(0.540)	(0.505)
Observations	464000	28500	71000	203000
Sample	all	0.5 bandwidth	bandwidth	2 bandwidth
**Panel B: Logit Model of Binary Y**				
treated10 = 1	0.0836	-0.0222	0.0392	-0.0125
	(p < 0.001)	(0.735)	(0.364)	(0.632)
firmsize_emp_rd10	0.0057	0.0415	-0.0068	0.0113
	(0.003)	(0.289)	(0.673)	(0.001)
1.treated10#c.firmsize_emp_rd10	-0.0056		0.0132	0.0028
	(0.003)		(0.526)	(0.652)
Observations	464000	28500	71000	203000
Sample	all	0.5 bandwidth	bandwidth	2 bandwidth
**Panel C: Probit Fitted Y**				
treated10 = 1	0.0004	-0.0005	-0.0008	-0.0004
	(0.182)	(0.721)	(0.374)	(0.505)
firmsize_emp_rd10	0.0013	0.0005	0.0012	0.0009
	(p < 0.001)	(0.532)	(p < 0.001)	(p < 0.001)
1.treated10#c.firmsize_emp_rd10	-0.0013		-0.0013	-0.001
	(p < 0.001)		(0.001)	(0.001)
Observations	464000	28500	71000	185000
Sample	all	0.5 bandwidth	bandwidth	2 bandwidth

Notes: 2018 ABS and 2016 LBD. Column (1) reports the global regression using all firms, column (2) restricts the sample on the half bandwidth, column (3) uses the full bandwidth, and column (4) uses two bandwidths. P-values are in parentheses.

### RDD power tests

RDD estimations yield statistically insignificant coefficients at the 10-employee cutoff, indicating no difference in reporting innovation activities around the cutoff. However, statistical inference based on insignificant results requires more elaboration [29], such as estimating the probability of detecting an effect if true, the effect size, and other parameters of the test, while taking into account the sample size. This is accomplished using RDD power tests [27] with the results presented in Table 4.

**Table 4:** RDD power tests.

Panel A: New-to-market Innovation					
Sampling BW	3.844				
Power against	H0: tau = 0	0.2*tau = 0.01	0.5*tau = 0.025	0.8*tau = 0.04	Tau = 0.05
Power value	0.05	0.492	0.998	1	1
Panel B: Fitted New-to-market Innovation					
Sampling BW	3.999				
Power against	H0: tau = 0	0.2*tau = 0.01	0.5*tau = 0.025	0.8*tau = 0.04	Tau = 0.05
Power value	0.05	1	1	1	1
Panel C: New-to-business Innovation					
Sampling BW	3.975				
Power against	H0: tau = 0	0.2*tau = 0.01	\\*tau = 0.025	0.8*tau = 0.04	Tau = 0.05
Power value	0.05	0.345	0.974	1	1
Panel D: Fitted New-to-business Innovation					
Sampling BW	3.995				
Power against	H0: tau = 0	0.2*tau = 0.01	0.5*tau = 0.025	0.8*tau = 0.04	Tau = 0.05
Power value	0.05	1	1	1	1

Notes: 2018 ABS. The RDD cutoff is 10 for employment size. Number of firms are 334,000 on the left of the cutoff and 130,000 on the right of the cutoff. When bandwidth is restricted, the effective number of firms are 40,000 on the left of the cutoff and 31,000 on the right of the cutoff.

Table 4 Panel A shows the power tests for the binary new-to-market innovation. We show some RDD power tests specifications in the Table notes. The full sample contains 334,000 observations (firms) below the cutoff and 130,000 observations above the cutoff, while the effective number of observations narrows down to 40,000 and 31,000 using the optimal bandwidth. The sample bandwidth is 3.844 for panel A, which means we use firms from which employment sizes are between 6 to 14 in the power calculations.

**Fig 6:**
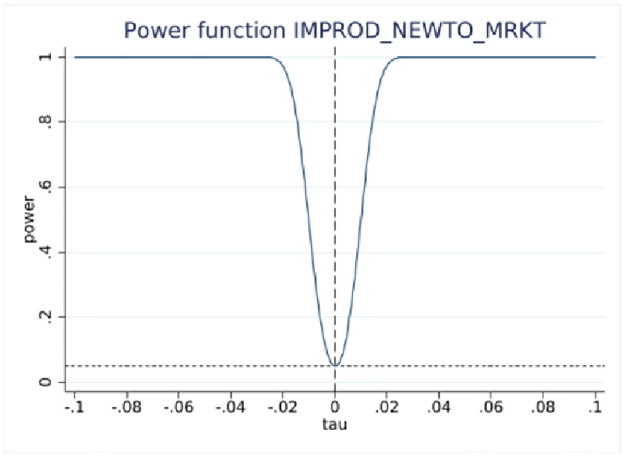
RDD power test for new-to-market innovation. Notes: ABS 2018 and LBD 2016, authors’ calculations. This figure corresponds to the power tests in Table 4 Panel A.

**Fig 7:**
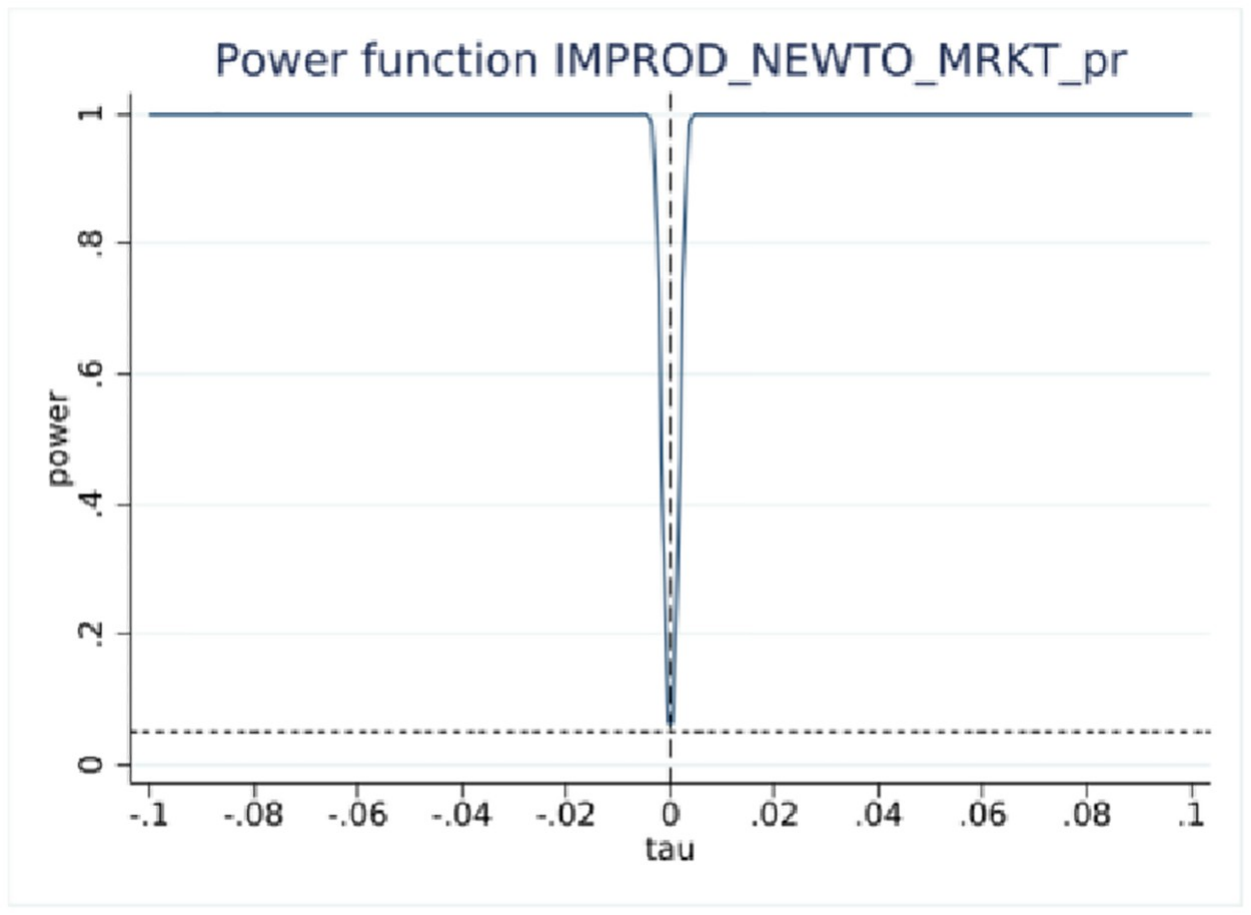
RDD power test for probit fitted new-to-market innovation. Notes: ABS 2018 and LBD 2016, authors’ calculations. This figure corresponds to the power tests in Table 4 Panel B.

**Fig 8:**
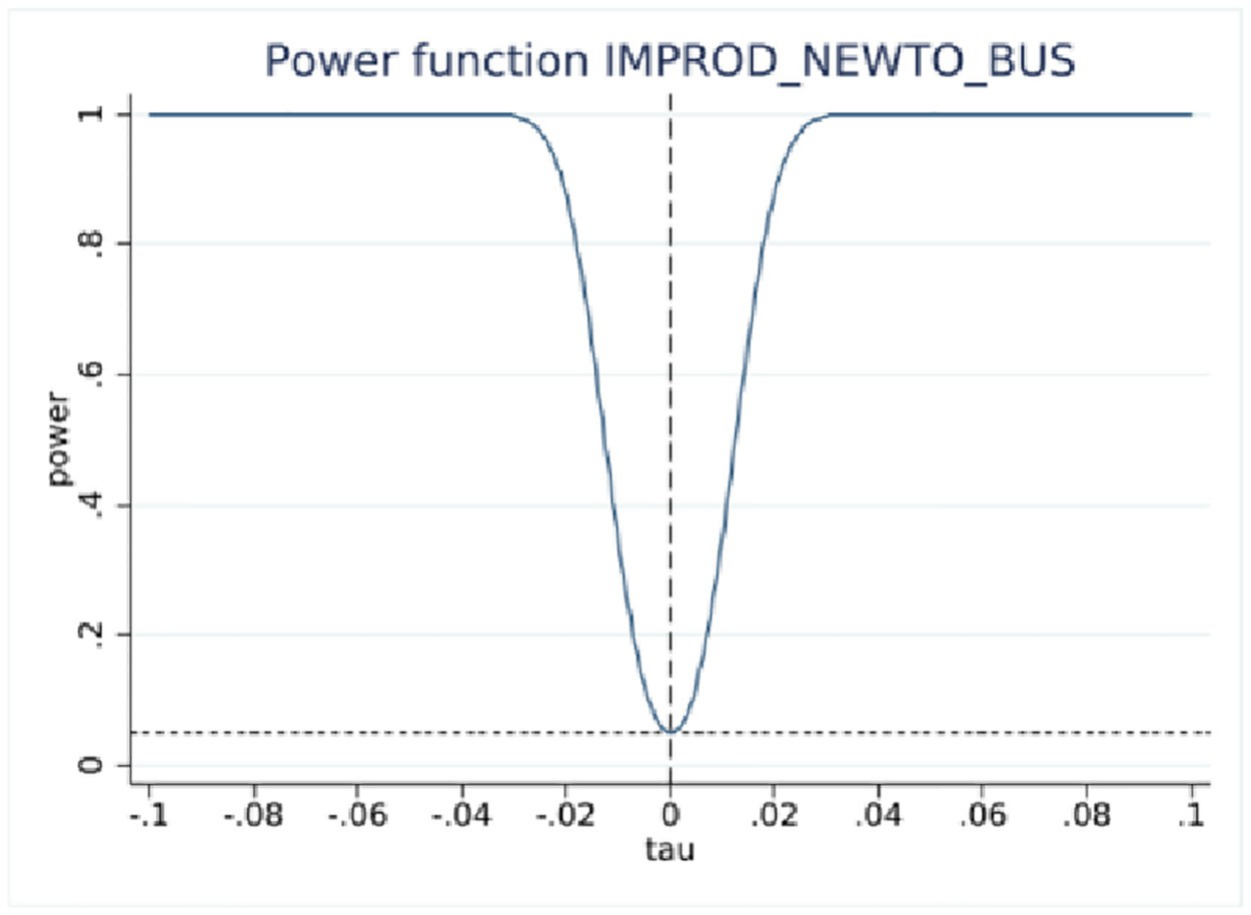
RDD power test for new-to-business innovation. Notes: ABS 2018 and LBD 2016, authors’ calculations. This figure corresponds to the power tests in Table 4 Panel C.

**Fig 9:**
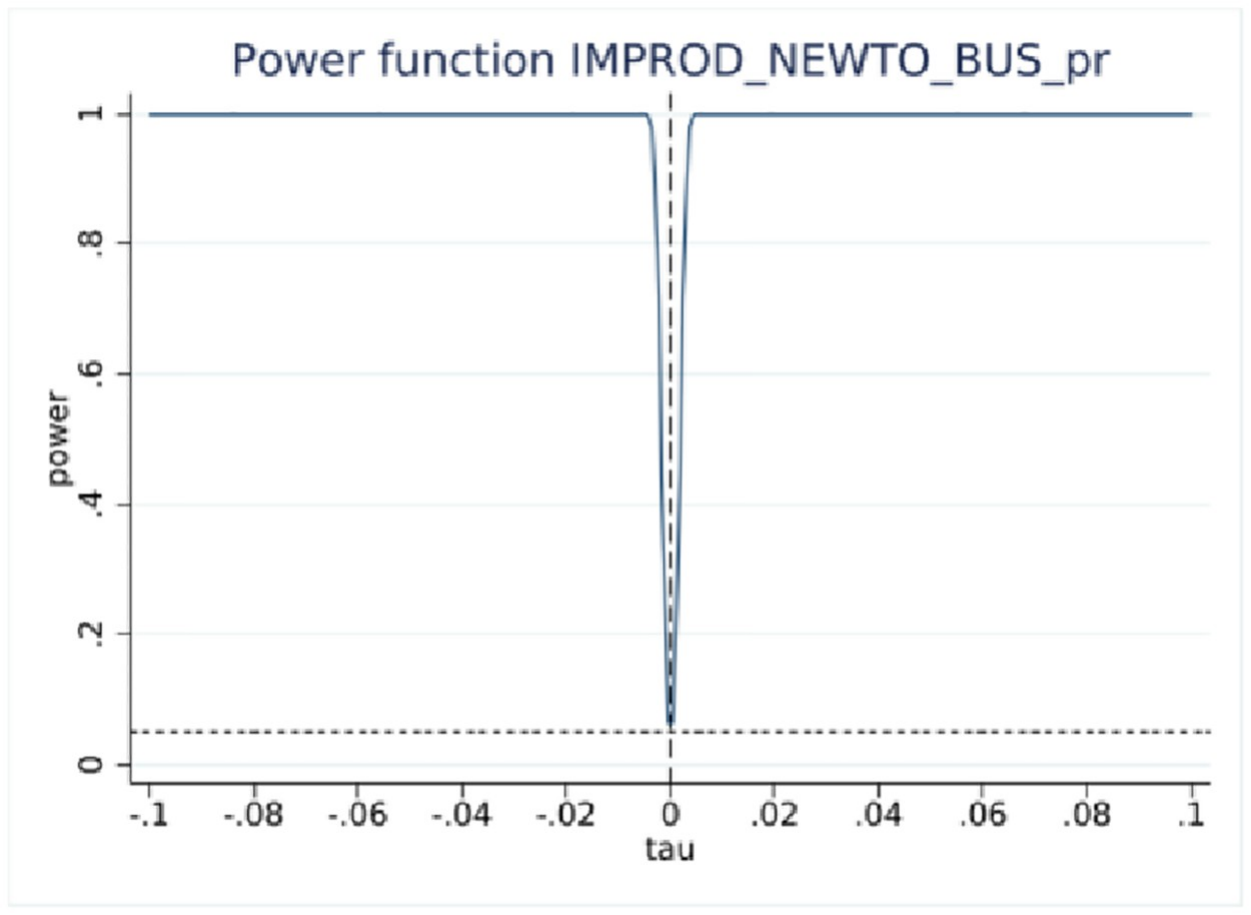
RDD power test for probit fitted new-to-business innovation. Notes: ABS 2018 and LBD 2016, authors’ calculations. This figure corresponds to the power tests in Table 4 Panel D.

One of the important parameters we set is τ, which “specifies the treatment effect under the alternative at which the power function is evaluated” [27]. The treatment effect is set at 0.05 that is roughly half the effect size derived from the analysis of BRDIS and ASE [5]. We are most interested in the power estimate for 0.5τ corresponding to an effect size that is one quarter of that observed in BRDIS. In Table 4 Panels A and C for the binary outcome innovation variables, we see that the power against the alternative of 0.5τ (0.025) is 0.998 and 0.974, respectively, while in Panels B and D using predicted values for new-to-market and new-to-business innovation, an effect size as small as 0.2τ (0.01) can be detected with near certainty. This statistical result adds additional credence to the preliminary visual evidence provided in Figs 2–5. Overall, the tests show our RDD results are estimated with a high degree of power against a reasonable range of alternatives, permitting the more definitive conclusion that our nonsignificant results can be interpreted as no bias in self-reported innovation by microbusinesses in the 2018 ABS. If the null result was due to measurement error, then this would be reflected in much lower statistical power. Figs 6–9 visually display the results of the power tests as shown in Table 4.

## Conclusion

Microbusinesses, which make up most of the employer firms in the United States and account for the largest share of the sample included in the ABS, have a large impact on the incidence of innovation reported in the country. Focusing on self-reported innovation activities in surveys, this study examines whether including an R&D module that only microbusinesses receive made these firms less likely to report innovation than firms with 10 or more employees. Past research identifies a significant downward bias in combined innovation-R&D surveys relative to innovation-only surveys [2, 5]. However, differences in survey design between ABS and earlier combined innovation and R&D surveys suggest this bias may have been ameliorated or eliminated. Using RDD tests at the sharp 10-employee threshold, we do not detect a statistically significant bias affecting microbusiness self-reports for either new-to-market or new-to-business product innovation, which is further confirmed with RDD power tests.

The U.S. is unique in fielding a large, nationally representative innovation survey that includes microbusinesses. Most EU countries administering the harmonized Community Innovation Survey limit coverage to firms with 10 employees or more, consistent with guidance from the Oslo Manual [1]. However, this resource has yet to be fully exploited by small business and innovation researchers who are seemingly unaware of R&D and innovation statistics for microbusinesses [30]. Finding that the lower incidence of innovation among microbusinesses is not attributable to survey design should be reassuring to small business researchers. The result gives more credence to analyses investigating alternative explanations for the seeming innovation lag of microbusinesses. Jankowski, Wojan, and Kindlon [31] provide evidence suggesting that the lower innovation rate by microbusinesses is driven by a large number of single-employee firms where the owner-operator does not have a college degree. Among nonperforming R&D microbusinesses that are not characterized in this way, innovation rates were nearly identical to those of small businesses with 10–49 employees. Han, et al. [32] confirm that the effect of cloud computing subscriptions on microbusiness innovation rates is similar to that of other firm size classes.

This analysis can also inform the choices within the Federal statistical system in moving to a single enterprise platform for the collection of business information. Plans for the inaugural ABS were released after the NASEM report on reengineering business surveys was drafted but before the report was published. The authors of the report “urge the Census Bureau to use the experience with the ABS to inform the development of our recommended A[nnual]B[usiness]S[urvey]S[ystem]” (National Academies of Sciences, Engineering, and Medicine 2020, p.159) [9]. Some of the attributes of 2018 ABS noted in this analysis that may have contributed to data quality in a survey where different respondents receive different modules include: 1) a neutral title; 2) sequencing of question modules from more general to more specific; 3) isolating respondents from potentially irrelevant questions (e.g., employment of PhD scientists and engineers) that may affect responses to relevant questions (e.g., introduction of a new or significantly improved product); and 4) reliance on electronic data collection. Survey mode may be the most important factor as web-based surveys can easily satisfy the requirement to effectively insulate respondents from potentially irrelevant questions in a manner that is difficult to accomplish with paper surveys. Paper surveys may also encourage strategic responses to skip over modules—e.g., an 8-employee firm reporting 10 employees in ABS to avoid responding to the R&D module—further reducing data quality and undermining the objectives of an integrated survey [8].

## Supporting information

S1 Appendix Definitions of innovation variables (2018 ABS). (DOCX)
