# Supplementary material for: Testing biasedness of self-reported microbusiness innovation in the annual business survey
Source: PLoS One. 2024 Jan 12;19(1):e0296667. doi: 10.1371/journal.pone.0296667 (PMC10786406; doi:10.1371/journal.pone.0296667)
Supplement: S1 Appendix — (DOCX) [file pone.0296667.s001.docx]

Appendix: Definitions of Innovation Variables (2018 ABS)

**New-to-market innovation:**

During the three years 2015 to 2017, were any of this business’s product innovations (goods or services): New to the market? This business introduced a new or significantly improved product (good or service) into your market before its competitors (it may have already been available in other markets). The dummy is equal to one if the answer to the above question is yes.

**New-to-business innovation:**

During the three years 2015 to 2017, were any of this business’s product innovations (goods or services): Only new to this business? This business introduced a new or significantly improved product (good or service) that was already available from its competitors in the market. The dummy is equal to one if the answer to the above question is yes.
